# Acute Kidney Injury Enhances Outcome Prediction Ability of Sequential Organ Failure Assessment Score in Critically Ill Patients

**DOI:** 10.1371/journal.pone.0109649

**Published:** 2014-10-03

**Authors:** Chih-Hsiang Chang, Pei-Chun Fan, Ming-Yang Chang, Ya-Chung Tian, Cheng-Chieh Hung, Ji-Tseng Fang, Chih-Wei Yang, Yung-Chang Chen

**Affiliations:** 1 Kidney Research Center, Department of Nephrology, Chang Gung Memorial Hospital, Taipei, Taiwan Chang Gung University College of Medicine, Taoyuan, Taiwan; 2 Chang Gung University College of Medicine, Taoyuan, Taiwan; University of Torino, Italy

## Abstract

**Introduction:**

Acute kidney injury (AKI) is a common and serious complication in intensive care unit (ICU) patients and also often part of a multiple organ failure syndrome. The sequential organ failure assessment (SOFA) score is an excellent tool for assessing the extent of organ dysfunction in critically ill patients. This study aimed to evaluate the outcome prediction ability of SOFA and Acute Physiology and Chronic Health Evaluation (APACHE) III score in ICU patients with AKI.

**Methods:**

A total of 543 critically ill patients were admitted to the medical ICU of a tertiary-care hospital from July 2007 to June 2008. Demographic, clinical and laboratory variables were prospectively recorded for post hoc analysis as predictors of survival on the first day of ICU admission.

**Results:**

One hundred and eighty-seven (34.4%) patients presented with AKI on the first day of ICU admission based on the risk of renal failure, injury to kidney, failure of kidney function, loss of kidney function, and end-stage renal failure (RIFLE) classification. Major causes of the ICU admissions involved respiratory failure (58%). Overall in-ICU mortality was 37.9% and the hospital mortality was 44.7%. The predictive accuracy for ICU mortality of SOFA (areas under the receiver operating characteristic curves: 0.815±0.032) was as good as APACHE III in the AKI group. However, cumulative survival rates at 6-month follow-up following hospital discharge differed significantly (*p*<0.001) for SOFA score ≤10 *vs*. ≥11 in these ICU patients with AKI.

**Conclusions:**

For patients coexisting with AKI admitted to ICU, this work recommends application of SOFA by physicians to assess ICU mortality because of its practicality and low cost. A SOFA score of ≥ “11” on ICU day 1 should be considered an indicator of negative short-term outcome.

## Introduction

Although there are currently numerous co-existing clinical scores for critically ill patients [sequential organ failure assessment (SOFA) [1], Simplified Acute Physiology *Score (SAPS)* [2], [3], Acute Physiology and Chronic Health Evaluation (APACHE) [4]–[6]], none of them has sufficient accuracy to predict outcome. Raising the sensitivity and specificity and increasing the number of parameters in order to enhance statistical power reduce the simplicity and cost effectiveness for clinical use. Given the aging population and numerous cases of co-morbidity in intensive care unit (ICU) setting today, acute kidney injury (AKI) remains a common and serious complication [7]–[9]. Pathophysiological factors associated with AKI are also incriminated in the failure of other organs, indicating that AKI is often part of a multiple organ failure syndrome. The occurrence of individual organ system failures varies among patients admitted to the ICU with AKI, with different degrees of association existing between individual organ system failures and ICU mortality. From this viewpoint, the SOFA score is an excellent tool for assessing the extent of organ dysfunction in critically ill patients with AKI [10], [11]. However, there is no extant literature comparing these scoring systems in the setting of AKI defined by the risk of renal failure, injury to kidney, failure of kidney function, loss of kidney function, and end-stage renal failure (RIFLE) classification in critically ill patients [12].

We hypothesized that the discriminative power of the SOFA score in predicting ICU mortality is further enhanced for patients with AKI compared to those without. Therefore, we undertook a post hoc analysis of a prospectively accumulated database, to explore 3 ICU mortality scoring systems (SOFA and APACHE II & III) in critically ill patients with/without AKI and to compare the scores in these heterogeneous groups in three ICU admission settings.

## Materials and Methods

### Study participants and data collection

This investigation was carried out at three ICUs of one tertiary-care referral center between July 2007 and June 2008. These ICUs included two medical ICUs and one coronary care unit (CCU). The Chang Gung Memorial Hospital Institutional Review Board approved the study and waived the need of informed consent because there was no breach of privacy and the study did not interfere with clinical decisions related to patient care (approval No. 101-3059B). The patient information was anonymized and de-identified prior to analysis. There were 885 admissions during this period; final diagnosis and admission duration were reviewed first. Patients were excluded if they stayed in the ICU for less than 1 day (n = 89) or had repeated ICU admission (n = 56). Patients under 18 years of age, with organ transplant and end-stage renal disease (ESRD) with long-term dialysis were excluded (n = 154). To determine the ICU outcomes, we also excluded patients admitted to the ICU for observation after invasive procedures (n = 43).

Finally, a total of 543 cases were enrolled in this study. Post hoc analysis of a prospectively accumulated database examined the demographics and clinical characteristics recorded on the first day of ICU admission.

### Definitions

Pertinent medical history included respiratory failure (need for mechanical ventilation), AKI (based on the RIFLE classification), severe upper gastrointestinal (GI) bleeding (defined as massive GI bleeding combined with shock or need of ventilator assistance for procedure), congestive heart failure (CHF, based on Framingham criteria and defined as New York Heart Association functional class IV), hepatic encephalopathy grade II (according to World Congresses of Gastroenterology), shock (defined as hypotension with systolic arterial blood pressure of 90 mm Hg despite adequate fluid resuscitation), severe sepsis (defined as presence of 2 or more systemic inflammatory response syndrome criteria, proven or suspected infection) and associated organ dysfunction (according to modified American College of Chest Physicians and Society of Critical Care Medicine consensus criteria), acute myocardial infarction (AMI, defined according to the 2007 Expert Consensus Document of Circulation from the European Heart Journal), acute respiratory distress syndrome (based on the American-European Consensus Conference), hepatic carcinoma rupture (diagnosed by image and ascites puncture), arrhythmia (defined as ventricular tachycardia, ventricular filtrations, 2- or 3-degree AV block) [12]–[16]. The worst physiological and biochemical values on the first day of ICU admission were recorded. Neurological scoring was not performed in patients who were paralyzed or sedated since their conditions were not classified as neurological failure. The best verbal response for in-patients who had been intubated but not sedated was determined according to clinical judgment. Illness severity was assessed by the following scoring systems: APACHE II, III and SOFA [1], [4], [5], [17]. The RIFLE was also evaluated at the time of ICU admission. Baseline serum creatinine (SCr) was the first value measured during hospitalization. The modification of diet in renal disease (MDRD) formula was used to estimate the baseline SCr levels in 45 patients because these patients had been admitted directly to the ICU and their previous SCr levels were unknown [12].

### Statistical analysis

Continuous variables were summarized by mean and standard error unless otherwise stated. The Kolmogorov-Smirnov test was used to determine the normal distribution for each variable. Student’s *t*-test was used to compare the means of continuous variables and normally distributed data; otherwise, the Mann-Whitney U test was used. Categorical data were tested using the Chi-square test or Fisher’s exact test. Furthermore, the Hosmer-Lemeshow goodness-of-fit test was used for calibration when evaluating the number of observed and predicted deaths in risk groups for the entire range of death probabilities. Discrimination was assessed using the area under a receiver operating characteristic curve (AUROC), which was compared by a nonparametric approach. The AUROC analysis calculated cutoff values, sensitivity, specificity, and overall correctness. Finally, cutoff points were calculated with acquiring the best Youden index. The index is defined as sensitivity+specificity −1, where sensitivity and specificity are calculated as proportions. Youden index has minimum and maximum values of −1 and +1, respectively, with a value of +1 representing the optimal value for an algorithm. Cumulative survival curves as a function of time were generated by the Kaplan-Meier approach and compared with a log rank test. All statistical tests were two-tailed; a value of *p*<0.05 was considered statistically significant.

## Results

### Subject characteristics

Five hundred and forty-three critically ill patients admitted to medical ICUs were enrolled. Table 1 shows demographic data, clinical characteristics and illness severity of patients with AKI and non-AKI. The median patient age was 62 years. Three hundred and ninety (71.8%) patients were males and 153 (28.2%) were females. Among these patients, one hundred and eighty-seven (34.4%) patients had AKI at ICU admission as determined by the RIFLE classification. Compared to non-AKI patients, the AKI group had a longer ICU stay, low hemoglobin (Hb) (*p*<0.001), less serum albumin (*p*<0.001), as well as more hospital and ICU mortality.

**Table 1:** Demographic data and clinical characteristics according to total v.s. AKI v.s. non-AKI.

	Total (n = 543)	AKI (n = 187)	Non-AKI (n = 356)	*p*-value	
Age (years)	62.2±0.7	64.1±1.1	61.6±0.9	NS (0.09)	
Male/Female	390/153	130/57	260/96	NS (0.359)	
Length of ICU stay (days)	11.7±0.5	13.7±1.0	10.7±0.7	0.009	
Length of hospital stay (days)	21.5±0.9	22.1±1.6	21.2±1.0	NS (0.60)	
Body weight on ICU admission (kg)	62.3±0.6	61.4±1.0	62.8±0.8	NS (0.27)	
GCS, ICU first day (points)	11±0.2	10±0.3	12±0.2	<0.001	
MAP, ICU admission (mmHg)	77.1±1.7	73.8±1.5	79.7±2.7	NS (0.083)	
Serum creatinine, ICU first day (mg/dl)	2.1±0.1	3.5±0.2	1.3±0.1	<0.001	
Arterial HCO_3_^−^, ICU first day	21.7±0.3	19.3±0.5	22.9±0.3	<0.001	
Serum sodium, ICU first day (mg/dl)	138.3±0.3	138.0±0.7	138.5±0.4	NS (0.545)	
Bilirubin, ICU first day (mg/dl)	4.3±0.4	6.6±0.8	3.1±0.3	<0.001	
Albumin, ICU first day (g/l)	2.8±0.1	2.5±0.1	3.0±0.1	<0.001	
Blood Sugar, ICU first day (mg/dl)	168.4±4.8	182.1±10.3	161±5.0	NS (0.073)	
Hemoglobin, ICU first day (g/dl)	10.4±0.1	9.7±0.2	10.8±0.1	<0.001	
Platelets, ICU first day (x10^3^/µL)	166.4±4.8	143.4±8.0	178.5±5.9	<0.001	
Leukocytes, ICU first day (x10^3^/µL)	10.9±0.3	11.9±0.7	9.7±0.4	0.005	
PaO_2_/FiO_2_, ICU first day (mmHg)	279.6±6.6	260.2±10.4	289.9±8.3	0.031	
ICU mortality (%)	220 (40.5)	116 (62.0)	104 (29.2)	<0.001	
Hospital mortality (%)	243 (44.8)	131 (70.1)	112 (31.5)	<0.001	
Need for renal replacement therapy (%)	34 (6.2)	34 (18.1)	–	–	
*Underlying diseases*					
Diabetes mellitus (%)	164 (30.2)	67 (35.8)	97 (27.2)	0.044	
Hypertension (%)	188 (34.6)	57 (30.4)	131 (36.7)	NS (0.135)	
Cardiovascular disease (%)	148 (27.3)	47 (25.1)	101 (28.4)	NS (0.421)	
Chronic kidney disease (%)	113 (20.8)	62 (33.1)	51 (14.3)	<0.001	
Liver disease (%)	191 (35.2)	74 (39.6)	117 (32.9)	NS (0.120)	
Malignancy (%)	105 (19.3)	35 (18.7)	70 (19.7)	NS (0.791)	
*Score systems*					
APACHE II, ICU first day (mean ± SE)	18.0±0.4	23.2±0.6	15.2±0.4	<0.001	
APACHE III, ICU first day (mean ± SE)	72.4±1.5	94.3±2.3	60.2±1.6	<0.001	
SOFA, ICU first day (mean ± SE)	7.4±0.2	10.5±0.3	5.8±0.2	<0.001	
RIFLE, ICU first day (mean ± SE)	0.8±0.1	2.0±0.1	–	–	
RIFLE-R (%)	66 (12.2)	66 (35.3)	–	–	
RIFLE-I (%)	54 (9.9)	54 (28.9)	–	–	
RIFLE-F (%)	67 (12.3)	67 (35.8)	–	–	

Abbreviation: AKI, acute kidney injury; NS, not significant; ICU, intensive care unit; SE, standard error; GCS, Glasgow coma scale; MAP, mean arterial pressure; PaO2, arterial partial pressure of oxygen; FiO2, fraction of inspired oxygen; APACHE, Acute Physiology and Chronic Health Evaluation; SOFA, sequential organ failure assessment; RIFLE, risk of renal failure, injury to the kidney, failure of kidney function, loss of kidney function, and end-stage renal failure.

Table 2 shows the demographic data and scoring systems of ICU survivors and non-survivors. In the non-survival group of the total population, these patients had longer ICU stays but fewer hospital days. In addition, low Hb (*p*<0.001) and serum albumin (*p*<0.001) were also noted in these patients. Moreover, the non-AKI ICU non-survival patients still had a significantly longer ICU stay but fewer hospital days. They also had very significantly lower Hb (*p*<0.001) and serum albumin (*p*<0.001) on ICU day 1. After adjusting the SOFA score in the multivariate analysis, the Hb and serum albumin still reached a significant difference (*p*<0.05) for those non-AKI patients. Furthermore, the AKI ICU non-survival patients had significantly fewer hospital days but not significantly shorter ICU stay. They also tended to have low Hb (*p* = 0.022) and serum albumin (*p* = 0.014) on the first day of ICU admission. However, the Hb and serum albumin were not found to be significantly different for these AKI patients after adjusting the SOFA score in the multiple logistic regression.

**Table 2:** Demographic data and clinical characteristics of total population, non-AKI and AKI groups according to ICU mortality.

	Total				Non-AKI group						AKI group		
	Survival(n = 337)	Non-survival(n = 206)	*p*-value		Survival(n = 266)		Non-survival(n = 90)		*p*-value		Survival(n = 71)	Non-survival(n = 116)	*p*-value
Age (years)	62.8±0.9	62.1±1.0	NS (0.616)		60.4±1.7		62.1±1.0		NS (0.396)		65.8±1.8	62.9±1.4	NS (0.223)
Male/Female	218/82	172/71	NS (0.802)		194/72		66/24		NS (0.981)		52/19	78/38	NS (0.387)
Length of ICU stay (days)	8.4±0.6	15.8±0.9	<0.001		8.7±0.7		16.7±1.6		<0.001		12.3±1.3	14.6±1.3	NS (0.241)
Length of hospital stay (days)	23.4±1.2	19.2±1.2	0.016		22.5±1.3		17.3±1.6		0.012		34.3±2.9	14.7±1.3	<0.001
Body weight on ICU admission (kg)	63.3±0.8	61.0±0.9	NS (0.055)		63.9±0.9		59.5±1.4		0.011		60.8±1.5	61.7±1.3	NS (0.666)
GCS, ICU first day (points)	12.7±0.2	9.9±0.3	<0.001		12.7±0.3		10.8±0.5		0.001		11.9±0.5	8.9±0.5	<0.001
MAP, ICU admission (mmHg)	80.1±3.8	75.1±1.4	NS (0.170)		81.7±4.3		76.1±2.4		NS (0.370)		80.5±2.7	71.3±1.8	0.008
Serum creatinine, ICU first day (mg/dl)	1.7±0.1	2.5±0.1	<0.001		1.7±0.1		1.8±0.2		NS (0.444)		2.9±0.6	2.7±0.4	NS (0.765)
Arterial HCO_3_^−^, ICU first day	22.8±0.4	20.2±0.4	<0.001		23.1±0.4		22.1±0.7		NS (0.220)		20.6±0.8	18.5±0.6	0.021
Bilirubin, ICU first day (mg/dl)	1.8±0.2	7.4±0.7	<0.001		2.3±0.3		5.2±0.8		0.001		2.1±0.6	9.3±1.1	<0.001
Albumin, ICU first day (g/l)	3.1±0.1	2.5±0.1	<0.001		3.2±0.1		2.5±0.1		<0.001		2.6±0.6	2.4±0.4	0.014
Hemoglobin, ICU first day (g/dl)	11.1±0.2	9.6±0.1	<0.001		11.2±0.2		9.7±0.3		<0.001		10.2±0.3	9.4±0.2	0.022
Platelets, ICU first day (x10^3^/µL)	186.8±6.1	141.7±7.4	<0.001		185.8±6.2		157.6±16.7		NS (0.077)		178.1±13.5	122.1±9.4	0.001
PaO_2_/FiO_2_, ICU first day (mmHg)	301.5±8.6	252.4±9.9	<0.001		308.2±9.3		233.9±16.7		<0.001		283.5±16.7	246.5±13.2	NS (0.086)
*Score systems*													
APACHE II, ICU first day (mean ± SE)	14.5±0.4	23.8±0.5	<0.001		13.0±0.4		21.0±0.7		<0.001		19.1±0.8	25.9±0.7	<0.001
APACHE III, ICU first day (mean ± SE)	54.3±1.5	94.1±1.9	<0.001		54.5±1.6		82.2±2.9		<0.001		75.5±2.6	106.6±2.8	<0.001
SOFA, ICU first day (mean ± SE)	5.3±0.2	10.1±0.3	<0.001		5.0±0.2		8.2±0.4		<0.001		7.7±0.4	12.2±0.3	<0.001
RIFLE, ICU first day (mean ± SE)	0.5±0.1	1.4±0.1	<0.001		–		–		–		1.5±0.1	2.3±0.1	<0.001

Abbreviation: AKI, acute kidney injury; NS, not significant; ICU, intensive care unit; SE, standard error; GCS, Glasgow coma scale; MAP, mean arterial pressure; PaO2, arterial partial pressure of oxygen; FiO2, fraction of inspired oxygen; APACHE, Acute Physiology and Chronic Health Evaluation; SOFA, sequential organ failure assessment.

The reasons for admission to the ICU are listed in Table 3. The major causes of ICU admission were respiratory failure (58%) and severe UGI bleeding (21.7%). The causes of AKI are listed in Table 4. The major causes of AKI are sepsis (34.2%), acute respiratory distress syndrome (ARDS) (17.1%) and hypovolemic shock (12.8%).

**Table 3:** Causes of ICU admission.

	All patients n (%)	Survivors n (%)	Non-survivors n (%)	*p*
Respiratory failure	315 (58.0)	152 (45.1)	163 (79.1)	<0.001
Cirrhosis with severe UGI bleeding	118 (21.7)	57 (16.9)	61 (29.6)	<0.001
CHF functional class IV	113 (20.8)	94 (27.9)	19 (9.2)	<0.001
Hepatic encephalopathy	108 (19.9)	42 (12.5)	66 (32.0)	<0.001
Shock	103 (19.0)	48 (14.72)	55 (26.7)	<0.001
Severe sepsis	103 (19.0)	33 (9.8)	70 (34.0)	<0.001
AMI	102 (18.8)	93 (27.6)	9 (4.4)	<0.001
ARDS	91 (16.8)	41 (12.2)	50 (24.3)	<0.001
HCC rupture	26 (4.8)	13 (3.9)	13 (6.3)	NS (0.194)
Arrhythmia	22 (4.1)	22 (6.5)	0 (0)	<0.001
Others^a^	22 (4.1)	14 (4.2)	8 (3.9)	NS (0.877)

Abbreviation: ICU, intensive care unit; UGI, upper gastrointestinal; CHF, congestive heart failure; AMI, acute myocardial infarction; ARDS, acute respiratory distress syndrome; HCC, hepatocellular carcinoma; NS, not significant;

a Acute pancreatitis severe form, cardiac tamponade, status epilepticus, pulmonary embolism, renal infraction.

**Table 4:** Causes of AKI.

	All AKI patients(n = 187)	AKI Survivors(n = 71)	AKI Non-survivors(n = 116)	*p*
Sepsis without shock	37 (19.8)	13 (18.3)	24 (20.7)	NS (0.692)
Sepsis with shock	27 (14.4)	6 (8.5)	21 (18.1)	NS (0.068)
Hypovolemic shock(UGI bleeding, HCC rupture)	24 (12.8)	12 (16.9)	12 (10.3)	NS (0.193)
Decompensated heart failure	12 (6.4)	9 (12.7)	3 (2.6)	0.011
AMI	15 (8.0)	11 (15.5)	4 (3.4)	0.005
ARDS	32 (17.1)	10 (14.1)	22 (19.0)	NS (0.390)
Hepatorenal syndrome	22 (11.8)	2 (2.8)	20 (17.2)	0.002
Contrast induced nephropathy	9 (4.8)	5 (7.0)	4 (3.4)	NS (0.304)
Others^a^	9 (4.8)	3 (4.2)	6 (5.2)	NS (1.000)

Abbreviation: AKI, acute kidney injury; NS, not significant; UGI, upper gastrointestinal; HCC, hepatocellular carcinoma; AMI, acute myocardial infarction; ARDS, acute respiratory distress syndrome.

a Acute pancreatitis severe form, cardiac tamponade, status epilepticus, pulmonary embolism, renal infraction.

### Hospital mortality, short-term prognosis and results of the scoring systems

**Figure 1:**
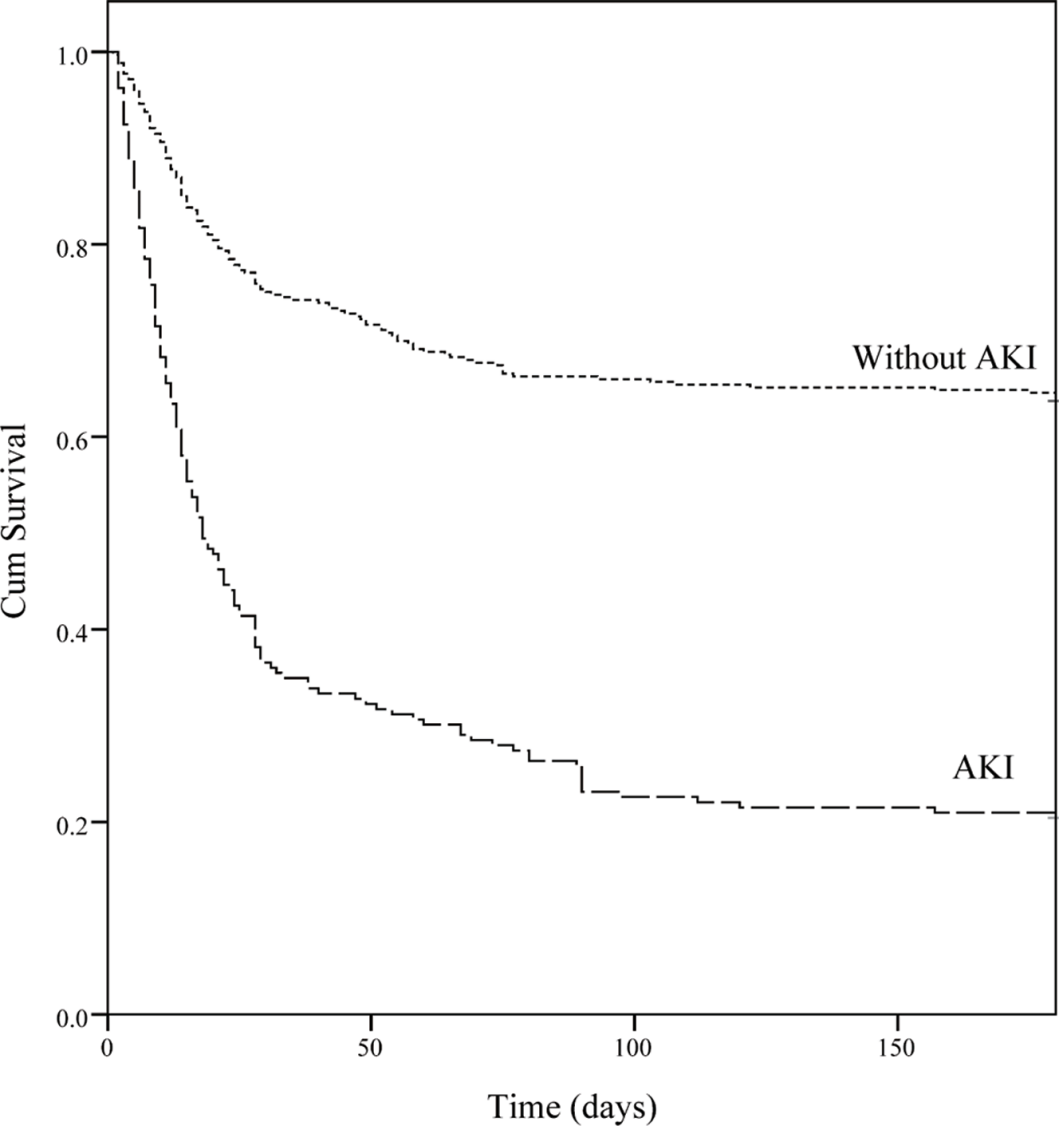
Comparison of cumulative survival between patients with and without acute kidney injury (AKI) (p<0.001).

**Figure 2:**
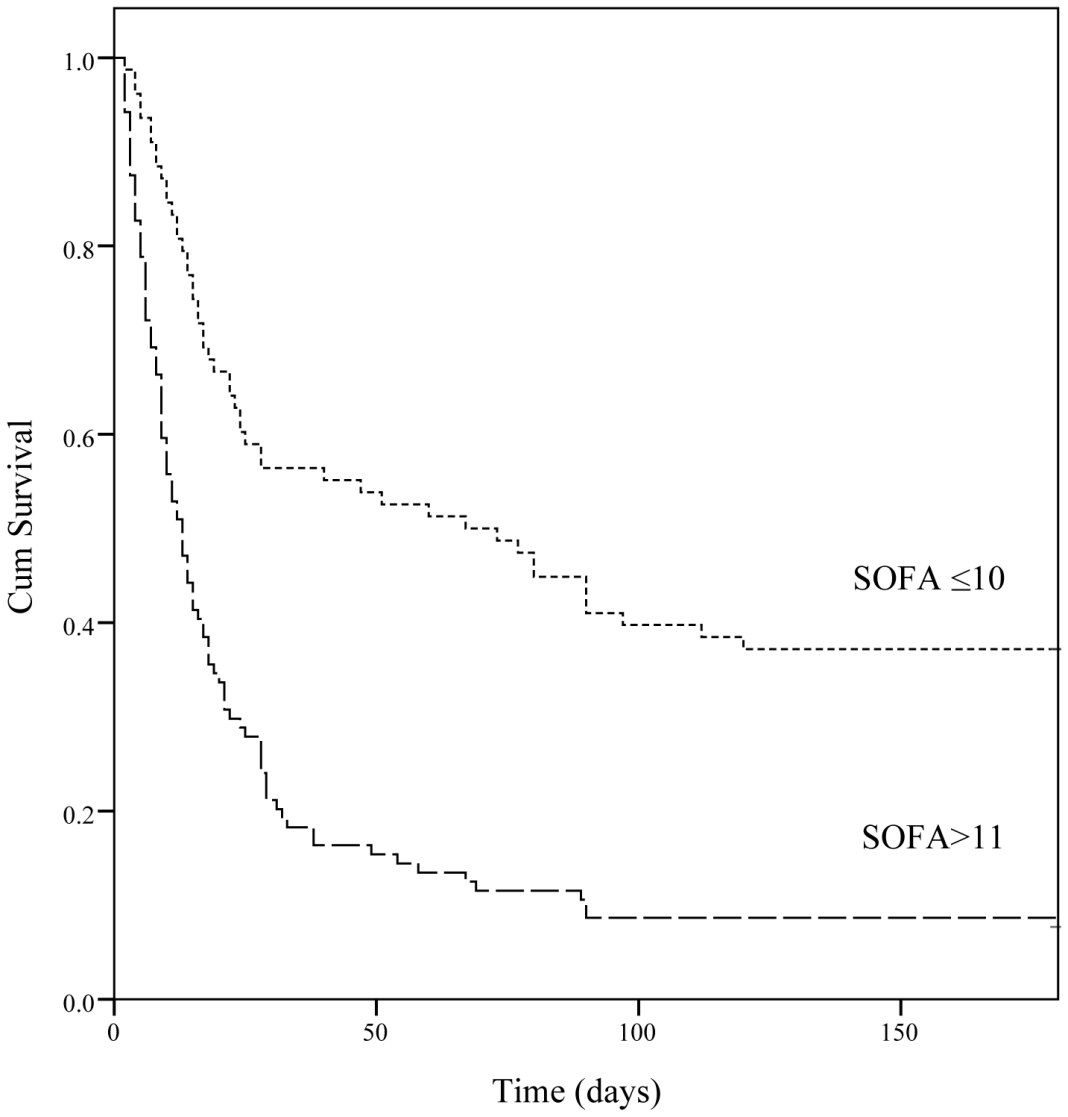
Cumulative survival for patients with coexisting AKI admitted to intensive care units were dichotomized at 10 SOFA points or less, and more than 11 SOFA points (p<0.001).

**Figure 3:**
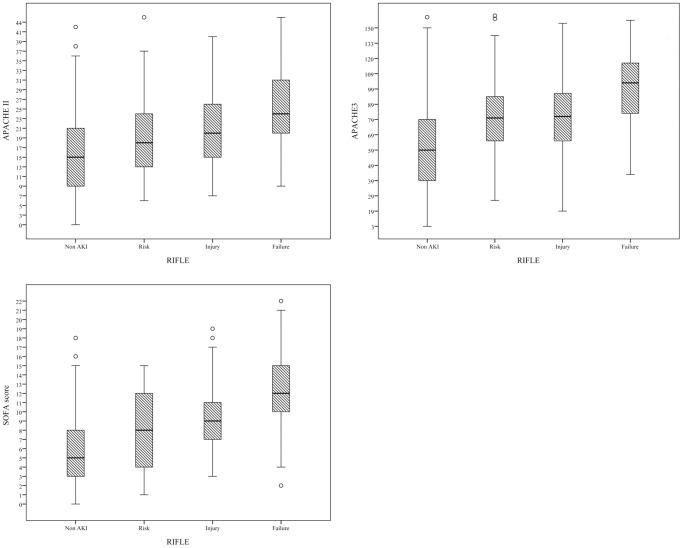
Box plot graph of APACHE II, APACHE III and SOFA score categorized by different AKI severity based on RIFLE classification. *P*<0.001 between groups by one-way analysis of variance (ANOVA).

Overall ICU mortality was 37.9% and the hospital mortality was 44.7%. As for the assessment of calibration, Table 5 lists goodness-of-fit, as measured by the Hosmer-Lemeshow analysis of predicted ICU mortality risk and the predictive accuracy of the APACHE III and SOFA scores in the different groups. In predicting the ICU mortality, the accuracy of SOFA was as good as that of APACHE III in the AKI group (AUROC: 0.815±0.032). In the non-AKI group, APACHE III was superior to SOFA (AUROC: 0.791±0.027 *vs*. 0.756±0.028). To assess the values of selected cutoff points for predicting ICU mortality, the sensitivity, specificity and overall correctness of prediction were determined (Table 6). The APACHE III scores revealed the best Youden index and highest overall correctness of prediction in both groups. In the AKI group, ICU mortality rates differed significantly (*p*<0.001) below and above cutoffs for 93 APACHE III points and 10 SOFA points. Cumulative survival rates at 6-month follow-up following hospital discharge differed significantly (*p*<0.001) for non-AKI vs. AKI groups (Fig. 1). Figure 2 shows the cumulative rates of survival for patients with coexisting AKI admitted to ICU were dichotomized at 10 SOFA points or less, and more than 11 SOFA points (*p*<0.001). To evaluate the correlation among APACHE II, APACHE III, SOFA score and various severity of AKI, we compared the means between groups according to the RIFLE classification by using ANOVA. The chi-square for trends revealed that the APACHE II, APACHE III, SOFA score were all significantly different between non-AKI and AKI groups with varying severity (*P*<0.001) (Figure 3).

**Table 5:** Calibration and discrimination for the scoring methods in predicting ICU mortality.

	Calibration			Discrimination		
	goodness-of-fit (χ^2^)	df	*p*	AUROC ± SE	95% CI	*P*
***Total***						
* APACHE II*	15.362	8	0.052	0.802±0.019	0.764 .839	<0.001
* APACHE III*	10.041	8	0.262	0.829±0.018	0.793–0.865	<0.001
* SOFA*	9.870	8	0.274	0.817±0.019	0.780–0.854	<0.001
* RIFLE*	5.690	2	0.058	0.703±0.025	0.655–0.752	<0.001
***AKI group***						
***AKI total***						
* APACHE II*	17.254	8	0.028	0.745±0.037	0.672–0.818	<0.001
* APACHE III*	4.800	8	0.779	0.815±0.032	0.752–0.877	<0.001
* SOFA*	7.263	8	0.508	0.815±0.032	0.751–0.878	<0.001
* RIFLE*	3.540	1	0.060	0.772±0.035	0.704–0.893	<0.001
***RIFLE-R***						
* APACHE II*	3.556	8	0.829	0.737±0.068	0.604–0.870	0.010
* APACHE III*	7.806	8	0.350	0.830±0.052	0.727–0.933	<0.001
* SOFA*	8.683	8	0.370	0.833±0.050	0.735–0.930	<0.001
***RIFLE-I***						
* APACHE II*	6.799	8	0.450	0.754±0.068	0.621–0.888	0.002
* APACHE III*	8.987	8	0.343	0.776±0.070	0.638–0.914	0.005
* SOFA*	8.944	8	0.347	0.787±0.069	0.651–0.923	0.003
***RIFLE-F***						
* APACHE II*	5.564	8	0.591	0.704±0.112	0.485–0.923	NS (0.121)
* APACHE III*	7.952	8	0.438	0.803±0.125	0.558–0.998	0.043
* SOFA*	3.602	8	0.824	0.832±0.064	0.707–0.956	0.008
***Non-AKI group***						
* APACHE II*	9.280	8	0.319	0.771±0.027	0.719–0.823	<0.001
* APACHE III*	8.080	8	0.426	0.791±0.027	0.739–0.843	<0.001
* SOFA*	11.725	8	0.164	0.756±0.028	0.702–0.810	<0.001

Abbreviation: ICU, intensive care unit; df, degree of freedom; AUROC, areas under the receiver operating characteristic curve; SE, standard error; CI, confidence interval; APACHE, Acute Physiology and Chronic Health Evaluation; SOFA, sequential organ failure assessment; AKI, acute kidney injury; RIFLE, risk of renal failure, injury to the kidney, failure of kidney function, loss of kidney function, and end-stage renal failure.

**Table 6:** Subsequent ICU mortality predicted after ICU admission.

Predictive Factors	Cutoff Point	Youden Index	Sensitivity (%)	Specificity (%)	Overall Correctness (%)
***Total***					
* APACHE II*	20^a^	0.46	88	56	72
* APACHE III*	80^a^	0.51	70	80	75
* SOFA*	7^a^	0.48	85	64	74
* RIFLE*	R^a^	0.34	93	34	64
***AKI group***					
* APACHE II*	21^a^	0.41	75	67	71
* APACHE III*	93^a^	0.49	68	82	75
* SOFA*	10^a^	0.47	75	73	74
* RIFLE*	I^a^	0.36	78	58	68
***RIFLE-R***					
* APACHE II*	24^a^	0.36	53	83	68
* APACHE III*	83^a^	0.56	90	67	79
* SOFA*	11^a^	0.45	74	67	71
***RIFLE-I***					
* APACHE II*	19^a^	0.46	75	71	73
* APACHE III*	99^a^	0.39	43	96	69
* SOFA*	10^a^	0.44	63	79	72
***RIFLE-F***					
* APACHE II*	24^a^	0.36	53	83	68
* APACHE III*	83^a^	0.56	90	67	79
* SOFA*	12^a^	0.61	67	100	81
***Non-AKI group***					
* APACHE II*	16^a^	0.44	85	57	71
* APACHE III*	52^a^	0.45	95	49	72
* SOFA*	7^a^	0.39	69	70	70

Abbreviation: ICU, intensive care unit; APACHE, Acute Physiology and Chronic Health Evaluation; SOFA, sequential organ failure assessment; AKI, acute kidney injury.

a Value giving the best Youden index.

## Discussion

This post hoc analysis of a prospectively accumulated database study included 543 heterogeneous patients with critical illnesses. The overall in-hospital mortality rate was 44.7%, which is consistent with figures obtained by previous studies indicating poor prognosis of ICU patients with septic shock, ARDS, AKI or hepatic cirrhosis. The occurrence of AKI in this work was 34.4% (187/543) and is in agreement with previous report indicating that the incidence was 35.8% of AKI (defined by the RIFLE classification) in a mixed ICU. The overall AKI group ICU mortality rate observed in this study was 62% (116/187), which was high compared to other studies [7]–[10], [18]. The high mortality may have been attributed to the inclusion of two hundred and fifty-two patients (46.4%) with underlying cirrhosis and ninety-one patients (16.8%) with ARDS, both of which are positively associated with mortality in AKI patients [19]–. In this study, we demonstrated that SOFA score at ICU day 1 has a comparable predictive ability for ICU mortality to APACHE III in the AKI group (AUROC 0.815 vs. 0.815). On the other hand, the APACHE III was superior to the SOFA score in the non-AKI group (AUROC 0.791 vs.0.756).

Renal failure is common in critically ill patients, and its occurrence is associated with an extremely high mortality rate [10], [12], [24], [25]. Therefore, outcome prediction for short-term prognosis is needed to identify patients with high mortality risk from AKI. Since ICU patients with AKI is a syndrome and often is precipitated by and co-existing with other organ failure, the SOFA scores performance in evaluating the function of multi-organ systems was found to be as good as those of APACHE III in this work. Beyond the AKI, the mortality in an ICU setting is often related to age and co-existing co-morbidity, thus making the APACHE III score more accurate. When facing AKI patients, assessing the function of other organs is important [10]. We can simply use the six parameters to predict the outcomes and apply this score to help clinicians to focus their attention on more severe patients. Given that the SOFA score ignores diagnosis, age and co-morbid conditions, this score probably reflects the unique characteristics of the present patient group, whose prognosis can be predicted without considering the factors of age and diagnosis. The influence of age on outcome has been demonstrated to decrease with an increase in disease severity [26], [27]. This can, at least partially, explain why age did not substantially influence the probability of mortality. The mortality rates greatly increased when SOFA scores of 11 or above were recorded in our study, which means that integrating the RIFLE classification and SOFA score, where ≥4 failed/dysfunction organs (including AKI), has fairly poor prognosis. Furthermore, researchers have come a long way in the study of the factors contributing to outcome in AKI patients. At present, the measure of AKI severity can be done using both ICU and specific AKI scores [11], [28]–[31]. Among the current general ICU scores, APACHE III and SOFA scores promise to be very useful. However, because the number and categorization of the variables considered in the APACHE III score is greater, it gains in statistical power but loses in simplicity, and thus the use of APACHE III entails extra cost [32].

In the non-AKI critically ill patients group, the discriminatory power of the APACHE III for predicting ICU mortality is superior to that of SOFA score. The non-AKI population in this study admitted to ICU was composed of a high proportion of subjects with AMI, CHF and cirrhosis. Anemia at baseline in patients with AMI undergoing primary percutaneous coronary intervention is common, and is strongly associated with adverse outcomes and increased mortality [33]–[35]. In CHF patients, low Hb values directly relate to poor peak oxygen consumption, disabling symptoms, and impaired survival [36], [37]. Das *et* *al*. retrospectively evaluated the outcomes of 138 ICU cirrhotic patients and identified the independent risk factors for in hospital mortality as age, albuminemia, international normalized ratio and the SOFA score computed after discarding points for hematologic failure [38]. As responses to chronic disease, causes cachexia and malnutrition (low Hb and albumin) as well as a systemic inflammation. A lack of Hb and serum albumin prognostic factors in the SOFA score may account for its discriminative inferiority to APACHE III.

Despite the promising analytical results of this study, several important limitations should be recognized. First, this study was conducted at a single institution; consequently, the results may not be directly extrapolated to other patient populations. Second, scoring was performed only on the first day of ICU admission. Sequential application of these scoring systems (for example, daily or weekly) may reflect the dynamic aspects of clinical diseases and thus provide more complete data for mortality risk. Third, the patient population was heterogeneous and contained a high proportion of AMI, CHF, ARDS and cirrhotic patients, and may be viewed as a special subgroup of critically ill patients. Further validation in other cohorts with a multicenter study may be required in the future research. Finally, use of the prognostic instruments in patients already admitted to ICUs rather than as a preadmission screening tool may have skewed measurement results.

Various novel biomarkers such as NGAL (Neutrophil gelatinase-associated lipocalin), KIM-1 (Kidney Injury Molecule-1), interleukin-18 and cystatin C have been developed to assist early diagnosis, differential diagnosis and prognostic prediction in patients with AKI [39], [40]. Combination of biomarkers and traditional scoring systems may further improve the sensitivity, specificity of clinical diagnosis and prognostic prediction, and remained to be clarified in the future.

## Conclusions

In conclusion, this investigation demonstrated that the prognosis for AKI patients admitted to ICU is poor. The analytic data also highlighted the good discriminative power of the SOFA score in predicting ICU mortality of critically ill patients with AKI defined by RIFLE classification. Considering the economy and ease of implementation, we suggest that SOFA score can increase the accuracy of short-term prognosis in this heterogeneous group of patients.

## References

[1] Vincent JL, Moreno R, Takala J, Willatts S, De Mendonca A, et al (1996) The SOFA (Sepsis-related Organ Failure Assessment) score to describe organ dysfunction/failure. On behalf of the Working Group on Sepsis-Related Problems of the European Society of Intensive Care Medicine. Intensive Care Med 22: 707–710. 8844239 10.1007/BF01709751

[2] Le GallJR, Lemeshow S, Saulnier F (1993) A new Simplified Acute Physiology Score (SAPS II) based on a European/North American multicenter study. Jama 270: 2957–2963. 8254858 10.1001/jama.270.24.2957

[3] Le GallJR, Loirat P, Alperovitch A, Glaser P, Granthil C, et al (1984) A simplified acute physiology score for ICU patients. Crit Care Med 12: 975–977. 6499483 10.1097/00003246-198411000-00012

[4] Knaus WA, Zimmerman JE, Wagner DP, Draper EA, Lawrence DE (1981) APACHE-acute physiology and chronic health evaluation: a physiologically based classification system. Crit Care Med 9: 591–597. 7261642 10.1097/00003246-198108000-00008

[5] Knaus WA, Wagner DP, Draper EA, Zimmerman JE, Bergner M, et al (1991) The APACHE III prognostic system. Risk prediction of hospital mortality for critically ill hospitalized adults. Chest 100: 1619–1636. 1959406 10.1378/chest.100.6.1619

[6] Keegan MT, Gajic O, Afessa B (2011) Severity of illness scoring systems in the intensive care unit. Crit Care Med 39: 163–169. 20838329 10.1097/CCM.0b013e3181f96f81

[7] Hoste EA, Clermont G, Kersten A, Venkataraman R, Angus DC, et al (2006) RIFLE criteria for acute kidney injury are associated with hospital mortality in critically ill patients: a cohort analysis. Crit Care 10: R73. 16696865 10.1186/cc4915 PMC1550961

[8] Barrantes F, Tian J, Vazquez R, Amoateng-Adjepong Y, Manthous CA (2008) Acute kidney injury criteria predict outcomes of critically ill patients. Crit Care Med 36: 1397–1403. 18434915 10.1097/CCM.0b013e318168fbe0

[9] Hsu RK, McCulloch CE, Ku E, Dudley RA, Hsu CY (2013) Regional variation in the incidence of dialysis-requiring AKI in the United States. Clin J Am Soc Nephrol 8: 1476–1481. 23929923 10.2215/CJN.12611212 PMC3805086

[10] de Mendonca A, Vincent JL, Suter PM, Moreno R, Dearden NM, et al (2000) Acute renal failure in the ICU: risk factors and outcome evaluated by the SOFA score. Intensive Care Med 26: 915–921. 10990106 10.1007/s001340051281

[11] Lin YF, Ko WJ, Wu VC, Chen YS, Chen YM, et al (2008) A modified sequential organ failure assessment score to predict hospital mortality of postoperative acute renal failure patients requiring renal replacement therapy. Blood Purif 26: 547–554. 19052448 10.1159/000178771

[12] Bellomo R, Ronco C, Kellum JA, Mehta RL, Palevsky P (2004) Acute renal failure - definition, outcome measures, animal models, fluid therapy and information technology needs: the Second International Consensus Conference of the Acute Dialysis Quality Initiative (ADQI) Group. Crit Care 8: R204–212. 15312219 10.1186/cc2872 PMC522841

[13] Thygesen K, Alpert JS, White HD, Jaffe AS, Apple FS, et al (2007) Universal definition of myocardial infarction. Circulation 116: 2634–2653. 17951284 10.1161/CIRCULATIONAHA.107.187397

[14] Ho KK, Anderson KM, Kannel WB, Grossman W, Levy D (1993) Survival after the onset of congestive heart failure in Framingham Heart Study subjects. Circulation 88: 107–115. 8319323 10.1161/01.cir.88.1.107

[15] American College of Chest Physicians/Society of Critical Care Medicine Consensus Conference: definitions for sepsis and organ failure and guidelines for the use of innovative therapies in sepsis. Crit Care Med 20: 864–874. 1597042

[16] Bernard GR, Artigas A, Brigham KL, Carlet J, Falke K, et al (1994) The American-European Consensus Conference on ARDS. Definitions, mechanisms, relevant outcomes, and clinical trial coordination. Am J Respir Crit Care Med 149: 818–824. 7509706 10.1164/ajrccm.149.3.7509706

[17] Knaus WA, Draper EA, Wagner DP, Zimmerman JE (1985) APACHE II: a severity of disease classification system. Crit Care Med 13: 818–829. 3928249

[18] Liu KD, Altmann C, Smits G, Krawczeski CD, Edelstein CL, et al (2009) Serum interleukin-6 and interleukin-8 are early biomarkers of acute kidney injury and predict prolonged mechanical ventilation in children undergoing cardiac surgery: a case-control study. Crit Care 13: R104. 19570208 10.1186/cc7940 PMC2750143

[19] du Cheyron D, Bouchet B, Parienti JJ, Ramakers M, Charbonneau P (2005) The attributable mortality of acute renal failure in critically ill patients with liver cirrhosis. Intensive Care Med 31: 1693–1699. 16244877 10.1007/s00134-005-2842-7

[20] Jenq CC, Tsai MH, Tian YC, Lin CY, Yang C, et al (2007) RIFLE classification can predict short-term prognosis in critically ill cirrhotic patients. Intensive Care Med 33: 1921–1930. 17605129 10.1007/s00134-007-0760-6

[21] Scott RA, Austin AS, Kolhe NV, McIntyre CW, Selby NM (2013) Acute kidney injury is independently associated with death in patients with cirrhosis. Frontline Gastroenterol 4: 191–197. 24660054 10.1136/flgastro-2012-100291 PMC3955898

[22] Cooke CR, Kahn JM, Caldwell E, Okamoto VN, Heckbert SR, et al (2008) Predictors of hospital mortality in a population-based cohort of patients with acute lung injury. Crit Care Med 36: 1412–1420. 18434894 10.1097/CCM.0b013e318170a375

[23] Lin CY, Kao KC, Tian YC, Jenq CC, Chang MY, et al (2010) Outcome scoring systems for acute respiratory distress syndrome. Shock 34: 352–357. 20844411 10.1097/SHK.0b013e3181d8e61d

[24] Liu KD, Matthay MA (2008) Advances in critical care for the nephrologist: acute lung injury/ARDS. Clin J Am Soc Nephrol 3: 578–586. 18199848 10.2215/CJN.01630407 PMC6631090

[25] Hsu CY, Chertow GM, McCulloch CE, Fan D, Ordonez JD, et al (2009) Nonrecovery of kidney function and death after acute on chronic renal failure. Clin J Am Soc Nephrol 4: 891–898. 19406959 10.2215/CJN.05571008 PMC2676192

[26] Rockwood K, Noseworthy TW, Gibney RT, Konopad E, Shustack A, et al (1993) One-year outcome of elderly and young patients admitted to intensive care units. Crit Care Med 21: 687–691. 8482089 10.1097/00003246-199305000-00011

[27] Van Den Noortgate N, Mouton V, Lamot C, Van Nooten G, Dhondt A, et al (2003) Outcome in a post-cardiac surgery population with acute renal failure requiring dialysis: does age make a difference? Nephrol Dial Transplant 18: 732–736. 12637642 10.1093/ndt/gfg043

[28] Abosaif NY, Tolba YA, Heap M, Russell J, El Nahas AM (2005) The outcome of acute renal failure in the intensive care unit according to RIFLE: model application, sensitivity, and predictability. Am J Kidney Dis 46: 1038–1048. 16310569 10.1053/j.ajkd.2005.08.033

[29] Ahlstrom A, Kuitunen A, Peltonen S, Hynninen M, Tallgren M, et al (2006) Comparison of 2 acute renal failure severity scores to general scoring systems in the critically ill. Am J Kidney Dis 48: 262–268. 16860192 10.1053/j.ajkd.2006.04.086

[30] Mehta RL (2003) Outcomes research in acute renal failure. Semin Nephrol 23: 283–294. 12838497 10.1016/s0270-9295(03)00064-0

[31] Chertow GM, Soroko SH, Paganini EP, Cho KC, Himmelfarb J, et al (2006) Mortality after acute renal failure: models for prognostic stratification and risk adjustment. Kidney Int 70: 1120–1126. 16850028 10.1038/sj.ki.5001579

[32] Chen YC, Tian YC, Liu NJ, Ho YP, Yang C, et al (2006) Prospective cohort study comparing sequential organ failure assessment and acute physiology, age, chronic health evaluation III scoring systems for hospital mortality prediction in critically ill cirrhotic patients. Int J Clin Pract 60: 160–166. 16451287 10.1111/j.1742-1241.2005.00634.x

[33] Anker SD, Voors A, Okonko D, Clark AL, James MK, et al (2009) Prevalence, incidence, and prognostic value of anaemia in patients after an acute myocardial infarction: data from the OPTIMAAL trial. Eur Heart J 30: 1331–1339. 19383732 10.1093/eurheartj/ehp116

[34] Aronson D, Suleiman M, Agmon Y, Suleiman A, Blich M, et al (2007) Changes in haemoglobin levels during hospital course and long-term outcome after acute myocardial infarction. Eur Heart J 28: 1289–1296. 17363447 10.1093/eurheartj/ehm013

[35] Hsu CY, Ordonez JD, Chertow GM, Fan D, McCulloch CE, et al (2008) The risk of acute renal failure in patients with chronic kidney disease. Kidney Int 74: 101–107. 18385668 10.1038/ki.2008.107 PMC2673528

[36] Maraldi C, Volpato S, Cesari M, Onder G, Pedone C, et al (2006) Anemia, physical disability, and survival in older patients with heart failure. J Card Fail 12: 533–539. 16952787 10.1016/j.cardfail.2006.05.002

[37] Groenveld HF, Januzzi JL, Damman K, van Wijngaarden J, Hillege HL, et al (2008) Anemia and mortality in heart failure patients a systematic review and meta-analysis. J Am Coll Cardiol 52: 818–827. 18755344 10.1016/j.jacc.2008.04.061

[38] Das V, Boelle PY, Galbois A, Guidet B, Maury E, et al (2010) Cirrhotic patients in the medical intensive care unit: early prognosis and long-term survival. Crit Care Med 38: 2108–2116. 20802324 10.1097/CCM.0b013e3181f3dea9

[39] Chen TH, Chang CH, Lin CY, Jenq CC, Chang MY, et al (2012) Acute kidney injury biomarkers for patients in a coronary care unit: a prospective cohort study. PLoS One 7: e32328. 22384218 10.1371/journal.pone.0032328 PMC3285210

[40] Han WK, Bailly V, Abichandani R, Thadhani R, Bonventre JV (2002) Kidney Injury Molecule-1 (KIM-1): a novel biomarker for human renal proximal tubule injury. Kidney Int 62: 237–244. 12081583 10.1046/j.1523-1755.2002.00433.x

